# Reduced eye optical quality contributes to worse chromatic thresholds in aging

**DOI:** 10.3389/fnint.2023.1129315

**Published:** 2023-03-24

**Authors:** Marcelo Fernandes Costa, Livia Soledade Rego, Leonardo Dutra Henriques, Carlo Martins Gaddi, Givago Silva Souza

**Affiliations:** ^1^Department of Experimental Psychology, Institute of Psychology, University of São Paulo, São Paulo, São Paulo, Brazil; ^2^Núcleo de Neurociências Aplicada, University of São Paulo, São Paulo, São Paulo, Brazil; ^3^Tropical Medicine Center, Federal University of Pará, Belém, Pará, Brazil

**Keywords:** aging, chromaticity threshold, ocular straylight, ocular optical quality, psychophysical

## Abstract

**Purpose:**

Aging causes substantial changes in the intraocular lens, which leads to a reduction in chromatic perception. We aimed to measure the ocular light dispersion component in relation to the reduction in color vision by aging.

**Methods:**

Intraocular straylight was quantified psychophysically by C-Quant for light dispersion [Log(s)], reliability of the results (ESD), and psychometric sampling quality (Q). The Cambridge Color Test Trivector protocol measured the chromaticity thresholds for protan, deutan, and tritan color confusion axis in CIE 1976 u’ v’ units. We tested 224 subjects aged 24–68 years (106 men) with normal best-corrected visual acuity and without clinical evidence of cataracts.

**Results:**

A significant positive correlation was found between ocular dispersion of light and chromaticity thresholds for protan (r = 0.42; *p* < 0.001), deutan (r = 0.49; *p* < 0.001) and tritan (r = 0.51; *p* < 0.0001) color confusion axes with a moderate effect size (η^2^ = 0.39). However, a weak contribution of the logarithm of the straylight in predicting the chromaticity threshold for protan (b = 0.15; *p* = 0.025), deutan (b = 0.27; *p* = 0.001) and tritan (b = 0.21; *p* = 0.001) color confusion axes was verified in the regression coefficients. The other two measurement quality parameters estimated in the C-Quant were not correlated with chromaticity thresholds, suggesting that there are no problems with the quality of the measurement performed.

**Conclusion:**

An increase in ocular light dispersion that occurs physiologically with aging negatively impacts the chromaticity threshold in a similar manner across all three color confusion axes. The weak regression effects suggest that neural rather than optical processes were more related to the reduction in chromaticity in aging.

## Introduction

Ocular media structures are highly impaired during aging. Several visual functions are impaired due to decreased optical quality by straylight, light scattering, diffraction in pupil edges, and other aberrations. In addition to optical degradation, retinal structures, and functions are also reduced. However, the relative contributions of retinal and optical factors are under debate.

The crystalline is strongly affected by aging, leading to reduced accommodation, an increase of ametropia, high-order aberrations, and optical distortions such as straylight and opacities. During physiological aging, the molecular changes that occur in the crystalline lens contribute to a constant and gradual reduction in transparency (Alio et al., 2005; Camprodon et al., 2010). As an important consequence, light scattering increases because biological particles (protein, cells, etc.) are present and increasingly accumulate in the aging eye, increasing the ocular media opacity. The amount of retinal straylight in a healthy young human eye was considered to originate from the cornea (33%), lens (33%), and iris, sclera, and fundus for the remaining percentage (Coppens et al., 2006; de Wit et al., 2006).

Ocular straylight is a functional intensity measurement of light spreading perception. Subjects with increased light dispersion may refer to symptoms as “blurred vision,” significantly reduced contrast sensitivity and visual acuity, and difficulty with high-level visual processing such as face recognition. They also often complain of optical effects when looking against the light, such as colored halos around bright lights (van den Berg et al., 2010). High-order aberrations and straylight optical changes directly degrade spatial information, which reduces the modulation transfer function (MTF) increasing the point spread function, and reducing visual resolution and contrast discrimination (Artal et al., 2003). As a direct consequence, visual acuity and contrast sensitivity function are reduced in aging (Alio et al., 2005; Allard et al., 2013). Recently, a negative correlation was found between the estimated straylight in the eye and the reading performance of healthy participants, suggesting that the reduction in visual performance was related to the changes in their individual optical properties (Longhi Bitencourt et al., 2019). The CIE (Commission Internationale de l’Eclairage) recently defined the ocular straylight as a visual handicap but with a more general nature, and consequently more significant, than those of the glare condition (van den Berg et al., 2013).

Color vision is also reduced by aging. This decrease is frequently observed for short-wavelengths due to age-related yellowing of the ocular media (Hardy et al., 2005). The yellowing of the crystalline has been considered the main factor for this specific reduction, since the macular pigment density is relatively stable across ages (Demirel et al., 2014). However, a study controlling monochromatic aberrations showed a reduction in chromatic appearance for small lights with wavelengths ranging from 510 to 618 nm, suggesting a change affecting the L- and M-cones (Gupta et al., 2010). Colorimetric purity is also affected by age, reducing sensitivity to saturation in older subjects for both L-M and S-channel (Kraft and Werner, 1999).

Chromaticity discrimination using color-defined stimuli buried in a presented dynamic luminance-contrast noise found increased linear thresholds after the age of 20 years for remaining lifespan. One percent per year for red-green and about 1.6% for blue-yellow color confusion axes have been calculated for aging chromaticity losses (Barbur and Rodriguez-Carmona, 2016). Similar percentages were obtained using other methods as Cambridge Color Test (Paramei, 2012) and chromatic grating detection, both showing chromaticity thresholds increasing with age for all color confusion axes.

Finally, the physics of light scattering shows a phenomenon, in which a very strong wavelength dependence occurs for small particles scatter light and follows a power law (λ^–4^–termed Rayleigh dependence). There is a high ocular straylight for 450 nm light which is reduced up to 565 nm light which in turn increases again for longer wavelengths. Since Le Grand′s seminal work (Le Grant, 1937), several experiments have attempted to delineate the wavelength dependence of retinal straylight without conclusive results. Recently, a study revisited the wavelength dependence of retinal straylight (Coppens et al., 2006). The authors found that retinal straylight is strongly wavelength-dependent, as eyes with less pigmentation add extra straylight on the long wavelength side, and additional straylight increases with aging for all wavelengths. Thus, depending on individual characteristics of pigmentation level and age, retinal straylight may affect wavelength dependence. For well-pigmented brown eyes, we should expect near- perfect Rayleigh dependence. For less-pigmented blue eyes, the addition of red dominated component is expected, reducing the Rayleigh dependence.

Considering the distinct effect of straylight for different wavelengths, we hypothesized that retinal straylight could affect chromaticity discrimination in aging asymmetrically, meaning that short- and long-wavelengths should be more affected than middle-wavelengths in aging. Thus, in this study, we looked at the optical aspect linked to visual aging and optical damage by measuring the retinal straylight for the foveal region and comparing it to the chromaticity thresholds obtained for the protan, deutan and tritan color confusion axis. Since macular pigment is stable over the years, we aimed to investigate whether the high sensitivity of the computerized color vision test could verify the lower thresholds of the deutan color confusion axis by aging, suggesting an optical rather than a neural effect on color perception.

## Materials and methods

### Subjects

Two-hundred and eighty-three participants (138 men and 145 women) were recruited. All with best-corrected visual acuity equal to or better than 20/20, as measured by the ETDRS–Tumbling E chart (Xenônio Rep. Prod., São Paulo, Brazil) and refractive error of up to1.5 spherical diopters. According to Rozema et al. (2010), there is an early increase in the straylight value for these patients. All participants had brown eyes to avoid additional straylight for long-wavelengths, which will increase light dispersion parameters. Absence of ophthalmologic and/or type 2 diabetes mellitus, rheumatoid arthritis, renal and systemic arterial hypertension, central nervous system and other chronically significant systemic diseases were also excluding conditions. Finally, smokers and congenital color blindness verified by the 38-plate version of the Ishihara pseudoisochromatic (Kanehara Trade Inc., Tokyo, Japan) were also excluded.

Two-hundred and twenty-four participants (106 men and 118 women) were included in the experiment. Participants aged 23–68 years were grouped into six age groups: Group 1, 23–26 (mean = 25.4; sd = 0.94; *n* = 61); Group 2, 27–30 (mean = 29.9; sd = 0.85; *n* = 39); Group 3, 31–40 (mean = 34.6; sd = 2.72; *n* = 46); Group 4, 41–50 (mean = 46.4; sd = 3.07; *n* = 37); Group 5, 51–60 (mean = 56.7; sd = 2.04; *n* = 26); and group 6, 60–68 (mean = 65.3; sd = 2.40; *n* = 15) (Supplementary Table 1).

The participants were instructed not to use any product on the face such as make-up, solutions, creams, and cosmetic oils, as they could alter lacrimal and conjunctive regularities. This study followed the principles of the 1964 Declaration of Helsinki and its revised versions and was approved by the Ethics Committee of Institute of Psychology of the University of São Paulo. All participants provided written consent and were assured of complete anonymity.

### Equipment

#### Ocular straylight measurement

The C-Quant (Oculus Optikgeräte, Wetzlar, Germany) was used for absolute retinal straylight measurements. A 2-alternative forced-choice (2AFC) psychophysical adaptive staircase called Compensation Comparison Method was applied (van den Berg et al., 2010, 2013). Stimulation comprises a 14° visual field perimeter. The central target stimulus was a hemisected circle with 2^°^ of central area. The straylight source came from a white light annulus with an inner edge at 7^°^ and an outer edge at 14^°^ from the central visual field, which corresponds to a mean scattering angle of 10^°^ from the central fovea. The LED light produced a light source with maximum luminance of 300 cd.m^–2^. The equipment could be adjusted for inclination of the ocular and could be angled between 35 and 55^°^ for the comfort of the participants. When necessary, refractive errors were corrected on the C-Quant apparatus using appropriate lenses. Subjects who wear contact lenses were instructed to stop wearing them for at least 7 days prior to testing for stabilization of corneal conformation irregularities. Light dispersion values were calculated as logarithmic straylight [Log(s)]. Higher values indicate more straylight in their eyes, consequently increasing sensitivity to glare. We also measured the reliability of the test outcome as the expected standard deviation (ESD). The limit value of ESD ≤ 0.08 was used for normality purposes, as in a large European-driver study the overall accuracy was 0.1 log units (van den Berg et al., 2010). The eye contact with ocular can lead to palpebral warp and affect optical quality. In view of this, head stabilization was avoided. The eyes were randomly chosen, and the fellow eye was occluded by ophthalmological patches (Oftan, AMP, São Paulo, Brazil).

#### Chromaticity measurement

Chromaticity discrimination of the protan, deutan and tritan color confusion axis was measured using the Cambridge Color Test (CCT v2.0–Cambridge Research Instruments, Rochester, UK—commercial version), run on a computer (DELL Dymension XTC–600). The stimuli were generated by a graphic card VSG 2/5 (Cambridge Research Instruments, Rochester, UK), which allows 15 bits of ultra-high resolution per color channel and a high-speed LUT animation. Stimuli were generated on a high-resolution CRT color monitor, Sony FD Trinitron model GDM-F500T9 (Sony Corporation, Tokyo, Japan). The monitor was set to 1,024 × 768 pixels at 100 Hz frame refresh. The CS-100 chroma meter (Konica Minolta, Tokyo, Japan) was used for calibration of the individual color guns. All evaluations occurred in a dark room with participants 3 m away from the monitor, after 5 min of adaptation.

The stimulus consisted of a Landolt “C” letter that differed in chromaticity from the single neutral background (coordinates 0.1977, 0.4689 u’ v’ units of CIE 1976 color space) presented for 3 s in a pedestal circle area of 7^°^ against a black background surrounding. Small patches ranging in size from 0.5 to 2 cm in diameter and six luminance levels (8, 10, 12, 14, 16, and 18 cd.m^–2^) were randomly distributed in the pedestal area. The Landolt C gap size corresponded to 1.25^°^ of visual angle, the outer diameter 5.4^°^ and the inner diameter 2.75^°^at 3 m (Regan et al., 1994). The use of small circle sizes and different luminance, consists of a spatial and luminance noise, aiming to avoid the influence of cues generated by differences in luminance or target contours that affect chromaticity discrimination.

### Procedures

#### Ocular straylight measurement

In order to measure the disability effect of the light dispersion, a straylight source was induced from the white light annulus flicker in the surround of the visual field. The central target area was split in two horizontal hemifields. The flickering phase of the annulus’ light was in counter phase with the compensation light presented in one of the hemifields of the central circle. Both the straylight and the compensations lights change intensity during the test. We recorded the subjects’ response by pressing two buttons, representing the left and right test fields. Thus, the subject’s task was to choose by pressing the corresponding button of the hemifield that appeared to flicker more strongly.

A Two Alternative Forced-Choice (2-AFC) task procedure was used to judge which side the flicker appears stronger. The evaluation consisted of two conditions of light decrease. In the initial condition, the stimuli intensities had a step size of 0.1 log units, except for the first step which was 0.3 log units. Using the psychophysical method of limits, light intensities were presented in descending order of straylight (Franssen et al., 2006). In the final condition, the stimuli were logarithmically spaced by 0.05 log units in a fixed interval defined by the approximated threshold measured on the initial condition. Also, the stimuli were presented in random order, according to the psychophysical method of constant stimuli (Treutwein, 1995) to increase the precision of the light dispersion measurement and reduce hysteresis. At the end of the measurement, we obtained three parameters to analyze, the Log(s)—logarithm of the straylight, the estimated standard deviation (ESD), and a quality factor for psychometric sampling (Q). For measurement reliability purposes, the ESD value must be less than 0.08 and the Q value should be greater than 1.0 (Cervino et al., 2008).

#### Chromaticity discrimination

The Landolt’s C letter was randomly presented and the C-letter opening could be presented in one of four positions: up, down, right and left, configuring a 4-Alternative Forced Choice strategy. Using the CT3 response box (Cambridge Research Instruments, Rochester, UK), the participant had to press one of the four corresponding buttons. The participants had up to 5 s after the presentation of the stimulus to respond. No response or responses after 8 s were considered as errors. Participants of the age groups 5 and 6 were given an additional 2 s to press the buttons, for a total time of 10 s.

An adaptative psychophysical staircase procedure was used for threshold determination, meaning that the forward and backward step size used followed a dynamic rule based on the participant responses [for more detailed information, see Regan et al. (1994), Ventura et al. (2003), Costa et al. (2006)]. The staircase for each color confusion axis started with a highly saturated chromaticity and changed along the mathematical vector that connects these chromaticity to the white background chromaticity. The direction of the change depended on the participants’ response: the C-letter chromaticity approached the background chromaticity for each correct response and went back, increasing the distance from the background every time there was an incorrect response or no response for 8 s (10 for elderly-groups). The chromaticity change along the vectors could range from 0.1100 to 0.0020 units of CIE 1976 u’ v’. The psychophysical measurement ended when eleven staircase reversals were completed. An automatic fitting routine calculated the threshold for each vector, considering the average of the last seven reversals chromaticity.

### Statistical analysis

Statistical analysis was performed using Statistica software package (StatSoft v.12, Inc., Tulsa, OK, USA). Full descriptive analyses were performed. Statistical differences among the age groups were verified using One-Way ANOVA. We also evaluated ANOVA effect size to characterize the overall magnitude of population effect. Fisher LSD test was used to determine in which age-groups there were significant differences in the analysis of variance setting. Since the number of subjects in different age groups was different, the Fisher LSD test was considered, as it is one of the least conservative *post-hoc* tests. Correlation between age, C-Quant parameters and the chromaticity thresholds were verified with Pearson Correlation Product of the Moment. The relationship between chromaticity thresholds and the best predictor C-Quant parameters were accessed by a Multiple Regression procedure.

## Results

**FIGURE 1:**
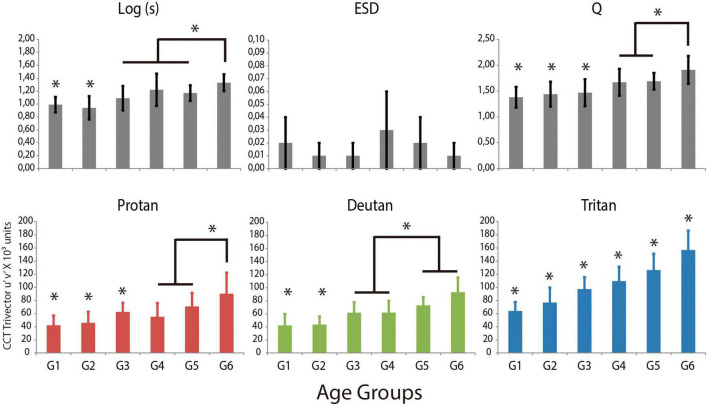
Presentation of the main results for the retinal straylight parameters and for the chromaticity discrimination thresholds for the protan, deutan and tritan color confusion axes in the CCT Trivector protocol. Statistical differences between age groups are marked (*). The Log(s) is the logarithm of the light dispersion; Q is the factor regarding the quality of the straylight curve; estimated standard deviation (ESD) is the expected standard deviation, which measures the reliability of the straylight.

A total of 224 subjects out of 283 (79%) met the inclusion criteria and were successfully evaluated. In addition, 8/224 (3%) of subjects had ESD values greater than 0.08 and were excluded from the analysis. The One-Way ANOVA test showed differences between the logarithm of the straylight with a moderate effect size (F = 20.54; *p* < 0.001; η^2^ = 0.39), in which Groups 1 and 2 had significant lower straylight [Log(s)] compared to other groups. Furthermore, Groups 3 and 4 were similar to Group 5, but these three had significant less dispersion compared to Group 6 (Figure 1—upper panels).

Chromaticity discrimination was also different between age groups for the protan color confusion axis, in which Groups 1, 2, and 3 had significant lower thresholds than other age groups, also Group 6 had higher thresholds than those of Group 4 and Group 5. For the deutan color confusion axis, Groups 1 and 2 had significant lower thresholds than other age groups; Groups 3 and 4 had showed lower thresholds than Groups 5 and 6. For the tritan color confusion axis, all age-groups had significant different thresholds (Figure 1—lower panels).

**FIGURE 2:**
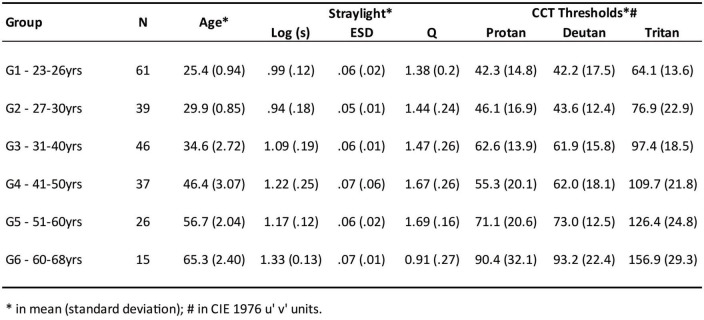
Bag plot showing the correlation between the amount of the straylight and the chromaticity thresholds measured in CIE u’ v’ units. Bag plots are the bivariate extension of the univariate box plot that contains 50% of the points similar to the 50% central region of the box-plot and a fence corresponding to an augmented central region by a factor of 1.5 (75% of data). Correlations measured were low-to-moderate for protan (r = 0.42; *p* < 0.001), deutan (r = 0.49; *p* < 0.001) and tritan (r = 0.51; *p* < 0.001) color confusion axes.

There are positive correlations between the logarithm of the straylight and the chromaticity thresholds for all three-color confusion axes—protan (r = 0.42; *p* < 0.001), deutan (r = 0.49; *p* < 0.001), and tritan (r = 0.51; *p* < 0.001). Bag plots are the bivariate extension of the univariate box plot with a bivariate depth region that contains 50% of the points similar to the 50% central region of the boxplot. The fence corresponded to an augmented central region by a factor of 1.5 (75% of data). In the Figure 2 we display a graphical bag-plot analysis of these correlations.

The magnitude of the standardized regression coefficients evaluates a weak contribution of the logarithm of the straylight in predicting the chromaticity threshold for protan (b = 0.15; *p* = 0.025), deutan (b = 0.27; *p* = 0.001) and tritan (b = 0.21; *p* = 0.001) color confusion axes.

The ESD and Q reliability parameters did not correlate to the chromaticity thresholds for any color confusion axis, evidencing good quality of the dispersion measure.

## Discussion

We successfully measured retinal straylight and chromaticity thresholds and obtained a moderate correlation, evidencing that aging-related reduction in chromatic discrimination is affected by the degradation of ocular media quality, increasing the dispersion and straylight for the light entering the eye. Additionally, the aging straylight evolution we assessed is similar to those previously reported (Rozema et al., 2010). Despite the moderate correlation measured the magnitude of the impact of straylight on chromaticity thresholds is definitively low. We infer from the result that the aging of the ocular media would not be the main effect in the worsening of the chromaticity discrimination measured here and by other studies evaluating aging on color vision discrimination.

The Rayleigh dependence shows high ocular straylight for short wavelengths light and for longer wavelengths, but lower ocular straylight for medium wavelengths (Le Grant, 1937). The dominant wavelength of the Cambridge Color Test protocol consist of 420 nm (tritan), 505 nm (deutan), and 660 nm (protan) (Schanda, 2015), which are similar to those used by Le Grant (1937). If these increases in ocular straylight at the extremes of visible light affected the measured chromaticity threshold, we would expect a stronger correlation between aging and chromaticity thresholds for the protan and tritan color confusion axes than for the deutan axis. However, our data show similar correlation for all three-color confusion axes and also similarly low magnitude for the contribution of straylight in increasing color thresholds for all axes. Chromatic threshold on the tritan axis was higher than protan and deutan, however, this trend is seen in most work with the Trivector mode of CCT (Costa et al., 2006, 2016; Paramei, 2012). Nevertheless, when observing the variation in age-related discrimination, there is no difference across the axis.

The lens turns yellowish with age due to the optical density of the lens that increases with age; besides that, there are residual components of fluorogens that act as a filter, particularly for the short wavelength end of the visible spectrum (Pierscionek, 1996). In fact, scattering and absorption, which are the main factors for the reduction in this short wavelength range, are related to the formation of high molecular weight protein aggregates, probably the derived insoluble protein, and accumulation of closely associated fluorogen (Krohn et al., 1981). One of the most important studies on lens transmissivity measured an increase in optical density (the inverse function for the Log of the light transmissivity), for spectral variation and aging effect (Weale, 1985). The author found an increase in optical density for light of wavelength shorter than 460 nm after 40–50 years, suggesting a strong short wavelength filtering due to lens yellowing.

Since optical quality is weakly related to reduced chromaticity in aging, the most likely hypothesis is that neural aspects must underlie this age-related color discrimination reduction. Indeed, changes in the morphology and physiology of retinal cells are likely causes of reduced color perception. Studies have shown that changes in retinal and choroidal tissues are intense in normal aging. Environmental factors also affect the retina over the years. Intense and frequent macular exposure to sunlight, which contains high levels of UV, generates mitochondrial changes already well-characterized in the literature. Briefly, such alterations occur in mitochondrial DNA, leading to alterations in ATP production, a key player in the retinoid cycle between photoreceptor and retinal pigment epithelium (RPE), culminating in apoptosis and cone death, mainly in the foveal region.

Macular pigment may be one of the sources of variation in reduced chromatic discrimination with aging. Certain dietary restrictions and changes in digestion mechanisms could reduce the availability of lutein and zeaxanthin, negatively impacting macular pigment optical density. However, a recent study shows that intra-subject and inter-subject variability is very high, therefore it is not a stable source of variability related to the reduced chromatic discriminability we measured. Thus, we are strongly inclined to consider the retina as the site that most affects the reduction of color discrimination with age.

It is also important to mention that the results we obtained in the Trivector protocol are similar to those found in previous studies. In this way, this work contributes to reinforce the value of the CCT to monitor changes in chromaticity discrimination due to aging. This information is of particular interest in studies of possible visual changes in elderly people with other vascular and neural diseases affecting retina such as aged-related macular disease (ARMD), choroidal neovascularization (CNV), glaucoma and diabetes.

## Conclusion

Our results support a relatively small effect of the physiological increase due to aging on ocular dispersion that negatively impacts the chromaticity thresholds. The negative correlation was similar for the three color confusion axes. Therefore, the higher increase in the chromaticity threshold in the blue-yellow axis (tritan) with increasing age must be mainly due to factors other than the elevated ocular dispersion caused by the increase in the opacity of the intraocular lens. Retina should be considered the primary site for the origin of age-related changes in measured color vision but as discussed, changes in ocular media pigmentation should play additional role in chromaticity changes.

## Data availability statement

The raw data supporting the conclusions of this article will be made available by the authors, without undue reservation.

## Ethics statement

The studies involving human participants were reviewed and approved by Comitê de Ética em Pesquisa com Seres Humanos do Instituto de Psicologia da USP. The patients/participants provided their written informed consent to participate in this study.

## Author contributions

MC, LR, and GS contributed to conception and design of the study. LR and LH organized the database. CM performed the statistical analysis. MC and CM wrote the first draft of the manuscript. MC, LR, GS, and LH wrote sections of the manuscript. All authors contributed to manuscript revision, read, and approved the submitted version.
